# Rapid imaging of peripheral vascular calcifications using in-phase, two-dimensional radial flash at 3 Tesla

**DOI:** 10.1186/1532-429X-18-S1-P366

**Published:** 2016-01-27

**Authors:** Robert R Edelman, Marcos P Ferreira Botelho, Amit Pursnani, Shivraman Giri, Ioannis Koktzoglou

**Affiliations:** 1 Radiology, NorthShore University HealthSystem, Evanston, IL USA; 2 Radiology, Feinberg School of Medicine, Northwestern University, Chicago, IL USA; 3 Radiology, Pritzker School of Medicine, University of Chicago, Chicago, IL USA; 4 Siemens Healthcare, Chicago, IL USA

## Background

The presence of dense peripheral vascular calcifications has negative prognostic implications in patients with peripheral arterial disease. In addition, the presence of dense calcifications may alter the choice of peripheral access site for patients undergoing percutaneous revascularization or TAVR procedures. Peripheral MR angiography is commonly used as an alternative to CT angiography for the evaluation of patients with PAD. While peripheral vascular calcifications are readily depicted with CT angiography, they are inapparent with MR angiography. We have previously demonstrated the ability of tailored 3D acquisitions to detect peripheral vascular calcifications [1]. However, these acquisitions are time-consuming and sensitive to patient motion. In order to overcome these limitations, we developed an alternative 2D approach using an in-phase radial FLASH pulse sequence. The technique was evaluated it in patients with peripheral vascular calcifications, using CT as the reference standard.

## Methods

The study was approved by the institutional IRB. Patients were recruited based on the presence of dense ilio-femoral vascular calcifications on peripheral CT angiography. Imaging was performed on a 3 Tesla MAGNETOM Verio (Siemens, Erlangen, Germany) MRI system using a prototype 2D spoiled gradient-echo (FLASH) pulse sequence, in-phase echo time of 2.46 ms, radial k-space trajectory with equidistant azimuthal view angle increments. Slice thickness was 2.1 mm, in-plane resolution 0.7 mm, inter-view spacing 5 ms, 2 signal averages, scan time per slice of 10 sec. A quiescent-interval slice-selective (QISS) non-contrast MR angiogram was used as the vessel localizer. Radial FLASH images were acquired in oblique coronal and axial planes through the ilio-femoral vessels and abdominal aorta during quiet breathing.

## Results

Aortic and ilio-femoral vascular calcifications appeared uniformly dark and contrasted with higher signal from the vessel lumen, fat, and muscle. The location and size of the calcifications showed excellent correlation with CT angiography. Free-breathing radial FLASH images of the abdominal aorta showed no ghosting artifact and sharply delineated the aortic wall calcifications.

## Conclusions

Peripheral vascular calcifications can be rapidly imaged using an in-phase, 2D radial FLASH technique. The use of a 2D sequential slice acquisition and radial k-space trajectory minimized sensitivity to respiratory and bowel motion, allowing evaluation of the aorta and pelvic arteries which is otherwise problematic using a 3D acquisition. Dark bands from chemical shift artifacts that can mimic low signal from vascular calcifications were absent due to the use of an in-phase radial acquisition.

**Figure 1:**
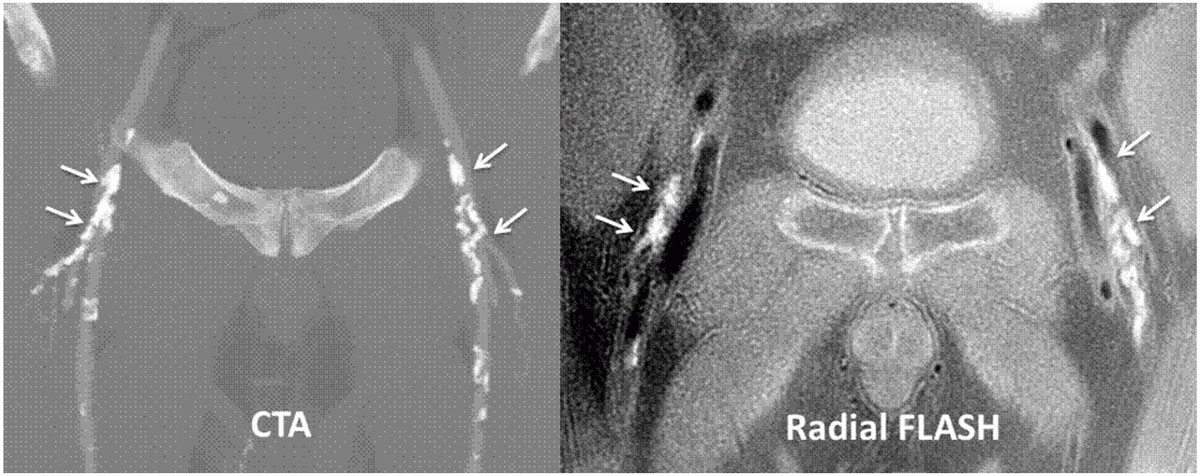
**Comparison of maximum intensity projection images from CT angiography (left) with minimum intensity projection images (right, displayed with inverted gray scale) from radial FLASH**. There is excellent correspondence in the appearance of the ilio-femoral vascular calcifications between the two imaging modalities.

## References

[1] Edelman RR (2015). Projection MR Imaging of Peripheral Arterial Calcifications. Mag Reson Med.

